# Workshop Results (coming soon)

**DOI:** 10.2196/jmir.2.suppl2.e10

**Published:** 2000-09-13

**Figure 1:**
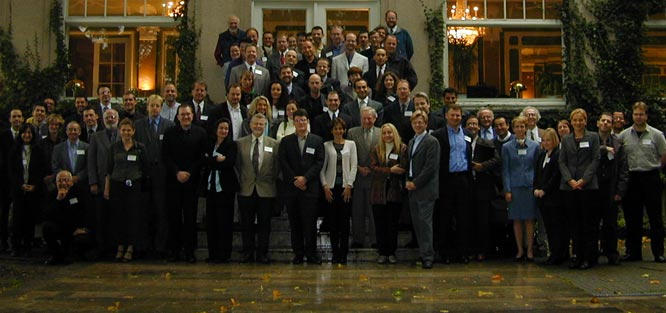
Photograph of workshop attendees.

More than 80 eHealth experts from 20 different countries and four continents attended an international workshop in Heidelberg/Germany to agree on an international scientific collaboration in the field of ehealth, to work on a metadata structure for ehealth providers and to discuss trustmark concept and other ways to enhance trust, implement eHealth ethics and improve the quality of health information on the Internet.

*List of attendees, in alphabetical order:*

Lucas M. Bachmann (Horten Zentrum für praxisorientierte Forschung und Wissenstransfer, Switzerland) Carl R. Blesius (MedCERTAIN, Germany), Markus Blume (mymedia GmbH, Germany), Carl J. Brandt (NetDoktor.com, Denmark), Dan Brickley (MedCERTAIN, United Kingdom), Alejandro Cacherosky (Buenos Aires Health Secretariat, Argentina), Ken Campell (Leukaemia Research Fund, United Kingdom), Richard Cleland (Federal Trade Comission, U.S.A.), Phil Cross (MedCERTAIN, United Kingdom), Elenice de Castro (PAHO/WHO Pan American Health Organization / World Health Organization, Brazil), Guy de Roy (Ordre des Medecins Conseil National, Belgium), Tony Delamothe (BMJ, United Kingdom), Martin D. Denz (Swiss Medical Informatics Association / Universitätsspital Zürich, Switzerland), Persephone Doupi (Erasmus University, Netherlands), Joan Dzenowagis (WHO World Health Organization, Switzerland), Christer Edling (The Swedish Society of Medicine, Sweden), Gunther Eysenbach (University of Heidelberg / MedCERTAIN, Germany), Gerard Freriks (TNO, Netherlands), Franz Frühwald (Internationales Büro der Österreichischen Ärztekammer, Austria), Lisa Gray (BIOME, United Kingdom), Pelle Gustafsson (Swedish Medical Association, Sweden), Gerhard Heine (European Commission, Luxembourg), Katrin Hörner (Arztpartner / Almeda, Germany), Robert Hsiung (University of Chicago, U.S.A.), Jostein Ingulfsen (Norwegian Board of Health, Norway), Thomas Isenberg (German Association of Consumer Organisations - Arbeitsgemeinschaft der Verbraucherverbände, Germany), Edward Jacob (KNMG, Netherlands), Alex R. Jadad (Canadian Cochrane Centre, Canada), Jacobo Kelber (ensalud.net, Mexico), Hugo Kitzinger (mymedia GmbH, Germany), Inge Kokot (Deutsches Grünes Kreuz e.V., Germany), Hans-Joachim Koubenec (Stiftung Warentest, Germany), Michel Labrecque (Université Laval Québec, Canada, Canada), Kristian Lampe (Finnish Office for Health Technology Assessment / MedCERTAIN, Finland), Stephane Lejeune (Swiss Cancer League, Switzerland), Leonard B. Lerer (INSEAD, France), Odile Leroy (PasteurMed, France), Nicolas Lienert (Medgate AG, Switzerland), Pål Lindström (LocusMedicus, Sweden), Sándor Lipp (Hungarian Ministry of Health, Hungary), Leena Lodenius (Finnish Duodecim Medical Society, Finland), Antti Malmivaara (Finnish Institute of Occupational Health, Finland), Miquel Angela Mayer Pujadas (Official Medical College of Barcelona, Spain), Peter Mills (EncycloMedica Ltd, United Kingdom), Cesar Molinero (Planet Medica, Belgium), Marc Muret (Zürcher Aerzte für Klassische Homöopathie, Switzerland), Wolfgang Nagel (GesundheitScout24 GmbH, Germany), Tim Nater (Health on the Net Foundation HON, Switzerland), Joerg Nitzsche (Zentralbibiliothek für Medizin Koeln, Germany), Debra O´Connor (Cochrane Consumer & Communication Group, La Trobe University, Australia), Gert Purkert (Arztpartner / Almeda, Germany), Ramon Sarrias Ramis (Official Medical College of Barcelona, Spain), Christine Reuter (A MedWorld AG, Germany), Ahmad Risk (Internet Healthcare Coalition, United Kingdom), Carl Bénédict Roth (GetWellness, Switzerland), Sebastian Schmid (Arztpartner / Almeda, Germany), Christiane Schmitz (Heinrich Heine Universität, Germany), Luk Schoonbaert (Belgacom Multimedia Ventures, Belgium), Frank Schuler (gfp Kommunikation Köln, Germany), Ulrich Schwanke (DocCheck, Germany), Sasha Shepperd (Imperial College School of Medicine, United Kingdom), Myra Sidrassi (MedCERTAIN, Germany), Chris Sigouin (McMaster University, Canada), Chris Silagy (Monash Medical Centre, Australia), Denise Silber (Internet Healthcare Coalition, U.S.A), Martin Sonderegger (Universitätsspital Zürich, Switzerland), Anke Steckelberg (Universität Hamburg, Germany), Frederik Tautz (Heinrich Heine Universität, Germany), Nicolas P. Terry (Center for Health Law Studies, St. Louis University, U.S.A.), Christian Thomeczek (Ärztliche Zentralstelle Qualitätssicherung, Germany), Wouter Tukker, (Praha Communications, Czech Republic), Gerard H. van der Zanden (The Netherlands Institute for Health Promotion and Disease Prevention NIGZ, Netherlands), Georg von Below (Swiss Medical Assoc./FMH, Switzerland), Frank von Danwitz (Deutsches Diabetes-Forschungsinstitut, Germany), C.-Peter Waegemann (Medical Records Institute, U.S.A.), Thomas Wetter (Dept. Of Medical Informatics, Universität Heidelberg, Germany), Petra Wilson, (European Commission, Belgium), Margaret A. Winker (JAMA, U.S.A.), Jeremy Wyatt (University College London, United Kingdom), Gabriel Yihune (MedCERTAIN, Germany)

